# Can the Marketing Authorisation Holder system improve the ESG performance of pharmaceutical manufacturers?

**DOI:** 10.3389/fpubh.2025.1587028

**Published:** 2025-06-11

**Authors:** Jiajing Li

**Affiliations:** School of Public Economics and Administration, Shanghai University of Finance and Economics, Shanghai, China

**Keywords:** Marketing Authorisation Holder system, ESG performance, R & D investment, internal pay gap, supply chain concentration

## Abstract

**Introduction:**

The Marketing Authorisation Holder (MAH) system is an advanced drug registration mechanism widely adopted globally Nevertheless, there is a scarcity of in-depth empirical investigations concerning MAH within the context of pharmaceutical manufacturing enterprises.

**Methods:**

This research endeavors to investigate the influence of MAH on the environmental, social and corporate governance (ESG) performance of companies through the application of the difference-in-differences model, leveraging firm data from China’s A-share-listed pharmaceutical manufacturing sector spanning from 2012 to 2019.

**Results:**

First, MAH markedly enhances the ESG performance of pharmaceutical manufacturing entities. Secondly, MAH impacts firms’ ESG performance via three pathways, namely, boosting research and development (R & D) investment, diminishing the internal pay disparity, and lowering supply chain concentration. Thirdly, the effect of MAH on firms’ ESG performance is more pronounced in non-state-owned firms, those with elevated management shareholding, and firms with enhanced internal control levels. Furthermore, this study ascertained that MAH exerted no influence on firm ESG variance and real earnings management.

**Discussion:**

The results offer actionable policy recommendations for refining the MAH system and promoting the sustainable development of pharmaceutical manufacturing firms. This study not only expands the research boundary of the economic consequences of the MAH system, but also affirms the key role of institutional innovation in the sustainable development of enterprises.

## 1 Introduction

Good health and medical conditions are fundamental to disease prevention, diagnosis, and treatment, playing a pivotal role in enhancing societal well-being. With the growing global population, aging population, and emphasis on health, the demand for medicines is increasing, especially innovative medicines. *PrecedenceResearch* data shows that innovative Active Pharmaceutical Ingredients (APIs) account for the largest share of the global active pharmaceutical ingredient market in 2022 (1). According to the data released by Frost and Sullivan, as of September 2024, the global innovative drug market has reached 1.7 trillion yuan. The COVID-19 pandemic underscored the critical importance of pharmaceutical innovation, reinforcing its role in addressing global health challenges.

Despite this growing demand, pharmaceutical manufacturers face significant hurdles, particularly in innovation and research and development (R & D). One of the key problems is the lack of innovation and the high cost of R & D in pharmaceutical manufacturing companies, which leads to high selling prices. As a result, the social benefits of pharmaceutical innovation are not high enough to generate positive externalities. According to *Evaluate Pharma* data, global pharmaceutical R & D expenses grew from $168.4 billion in 2017 to $203.9 billion in 2024, a Compound Annual Growth Rate (CAGR) of about 2.7%. The cost of R & D for individual new drugs has been on a significant upward trend over the past few decades. High drug prices have distorted the significance of R & D, which has led pharmaceutical manufacturing companies to emphasize more on financial benefits than health benefits (2).

Beyond financial challenges, pharmaceutical R&D and production pose severe environmental and ecological risks. Advances in medical technology have escalated the generation of medical waste, which, if improperly managed, can lead to environmental contamination and biodiversity loss (3). For instance, the incineration of biomedical waste contains polycyclic aromatic hydrocarbons (PAH) and high levels of heavy metals, which generate unfavorable amounts of hazardous substances that contaminate surface and groundwater (4). Beigaite et al. (5) contend that the existence of pharmaceutical substances and their movement in aquatic ecosystems have a considerable environmental impact worldwide.

To address these issues, scholars have explored strategies to enhance innovation performance in pharmaceutical enterprises. Gridchyna et al. (6) contends that the French Diagnosis Related Groups (DRG) paid funding fails to keep up with the development of innovative technologies or the progress of costly innovative medicines. Schuhmacher et al. (7) asserts that companies need to strengthen their core competencies in drug discovery and development, and they need to establish connections with academic partners and service providers to construct external networks and guarantee sustainable investment in R & D to produce a continuous flow of innovative drugs. Niwash et al. (8), relying on data from Jordanian pharmaceutical and medical supply companies, discovered that knowledge elements boost firms’ competitive edge through mechanisms like business intelligence, innovation speed, and innovation quality, and that high levels of human capital, relational capital, and structural capital. Li and Xu (9) discover that the policy of centralized volume-based drug procurement compels pharmaceutical firms to increase innovation inputs and enhances the quality of innovation outputs by exerting existential pressure on them.

In this context, China’s Marketing Authorization Holder (MAH) system represents a significant institutional innovation. Piloted in 10 provinces in November 2015 and implemented nationwide in December 2019, MAH decouples drug marketing authorization from production authorization. Before the implementation of MAH, China linked the marketing authorisation of medicines with the production authorisation, and the approval number of a medicine was only granted to the medicine manufacturer. Under this system, drug developers lacking their own production capacity had to either cooperate with a production-qualified company or transfer their R & D results to a manufacturing company. This hindered developers from obtaining the full value of their innovations. Correspondingly, the manufacturing company bore full responsibility for the quality of the drug, resulting in inadequate attention being given to the quality and safety of the drug in the R & D process. The binding of registration and production has caused overcapacity in some large-scale production firms. At the same time, some small innovative firms have difficulty in converting their R & D results into actual products due to their inability to obtain production licenses. The implementation of the MAH system aims to break through these constraints and facilitate the innovative development of the pharmaceutical industry.

This study investigates whether China’s MAH system enhances the Environmental, Social, and Governance (ESG) performance of pharmaceutical manufacturers.

This study is driven by the following aspects. First, as the world’s second-most populous nation with a rapidly aging demographic, China’s pharmaceutical sector sustainability is critical to global health. The annual total number of deaths in China is approximately 10 million, and in 2023, China’s pharmaceutical market sales exceed $2 trillion. If MAH is effective, it will contribute significantly to improving the health security of approximately 17.4 percent of the world’s population and provide a reference experience for many more countries. Secondly, in China, institutional problems are one of the key reasons for the dilemma of drug innovation (10), which are similar to those in many countries. China’s previous drug review and approval process was cumbersome and time - consuming. If China can promote R & D innovation and ESG performance of Chinese pharmaceutical manufacturing companies through institutional innovation, it will furnish guidelines for emerging economies across the globe. Thirdly, China’s pilot execution of MAH in 10 provinces from 2015 to 2019 furnishes us with a splendid research opportunity. We employ it as a quasi-natural experiment to probe into the effect of the MAH system on the ESG performance of pharmaceutical manufacturing firms through the difference-in-differences (DID) model.

Leveraging panel data from Chinese A-share listed pharmaceutical firms, we employ a difference-in-differences (DID) model to assess MAH’s impact on ESG performance and its underlying mechanisms. Our study has three main contributions. First, we concentrate on investigating the influence of MAH on the ESG performance of Chinese pharmaceutical manufacturing enterprises, augmenting the pertinent research on the efficacy of MAH. China’s MAH was rolled out nationwide in 2020, over 4 years ago, yet empirical evidence regarding its effectiveness remains scant. Presently, there is merely a limited amount of literature that examines the function of MAH on R & D innovation via empirical tests (11, 12), and we broaden the discourse on its impact. We discover that MAH propels pharmaceutical manufacturing firms in the pilot region to enhance their ESG performance.

Second, we decipher the ‘black box’ of the connection between MAH and firms’ ESG performance from the viewpoints of R & D, remuneration, and supply chain management, and probe into the impact mechanism, which enables us to gain a more lucid comprehension of the policy logic behind the positive impacts of MAH, and assists us in putting forward relevant insights for firm management. Our findings imply that MAH spurs pharmaceutical manufacturing firms to augment R & D investment, narrow internal pay disparities, and decrease customer concentration, but the effect on supplier concentration is not significant.

Third, based on a series of findings, we provide not only insights for pharmaceutical manufacturing firm managers, but also recommendations for policy - makers. Although these recommendations come from the Chinese scenario, as we mentioned earlier, China, as the second - largest country in terms of population and a representative emerging country, these recommendations may be relevant for many countries.

## 2 Literature review and hypothesis

### 2.1 Literature review

#### 2.1.1 Research on MAH

The MAH system represents a more comprehensive drug registration framework compared to traditional models that bundle marketing and manufacturing licenses. Central to the MAH system is the separation of these licenses, granting greater independence to holders of marketing authorizations (13).

The MAH system has been widely promoted globally, especially in European countries such as Germany, Italy, and Israel (14–18). However, its implementation has revealed variations in regulatory compliance and effectiveness. For instance, Handa et al. (19) found that while MAH in Japan mandated adverse event (AE) reporting, communication of these events to healthcare providers was often inadequate. Conversely, Alsaleh and Alshammari (20) asserted that MAH in Saudi Arabia distributed direct healthcare professional communication (DHPC) letters to guidelines with a satisfactory level of compliance.

In 2015, China initiated a pilot program for the MAH system, prompting scholarly exploration of its implications. China’s MAH will bring great changes and effects to the drug technology transfer system, drug commissioned production system, drug business licensing system, adverse drug reaction monitoring and pharmacovigilance system, drug damage liability system and other supporting regulatory systems (21). Based on the inter - provincial panel data of China’s pharmaceutical manufacturing industry, Liu et al. (11) used the synthetic control method to detect that MAH has a remarkable positive influence on the quality of innovation in pilot provinces. Based on data from A - share pharmaceutical manufacturing listed companies, Wan et al. (12) used the DID model to ascertain that MAH significantly boosted innovation inputs and outputs of pharmaceutical companies. However, MAH in China still has some flaws in promoting new drug development. In particular, drugs in China’s MAH are mainly chemicals - based, with most dosage forms being tablets and injections, and the amount of drugs varies widely across provinces (22).

While existing research has established the theoretical relationship between MAH and pharmaceutical companies, empirical evidence remains limited. On the one hand, empirical researches on MAH are scarce in quantity, both for China and other countries. On the other hand, these scarce empirical studies have merely concentrated on the influence of MAH on pharmaceutical innovation. Therefore, more empirical evidence is required to investigate the various impacts of MAH on pharmaceutical manufacturing companies.

#### 2.1.2 Research on ESG performance of pharmaceutical manufacturing firms

In recent years, firm ESG performance has garnered increasing attention from investors, prompting a growing body of scholarly research. The pharmaceutical manufacturing sector has numerous environmental, social and governance issues that pose challenges to its long-term sustainability (23), thus research on its ESG performance is of great significance.

The literature has approached this topic from two main perspectives. First, numerous studies have identified and analyzed the key dimensions of ESG performance in pharmaceutical manufacturing. Yu et al. (24) put forward a comprehensive MCDM framework for assessing the ESG sustainability performance of listed companies and discovered that the most crucial criteria in environmental, social, and governance aspects were pollution treatment, health and safety, and risk management. Lee et al. (25) carried out an online survey of 1,298 respondents. Two categories of firm social responsibility (CSR), promotion of public health and emergency disaster relief support, had the highest preference. Bae et al. (26) found that pharmaceutical companies in South Korea complied with ethical, legal, and economic responsibilities only, but did not contribute enough beyond these, such as innovative drug development.

Second, empirical studies have substantiated the strategic importance of ESG performance for pharmaceutical manufacturers. Based on Fuzzy - set Qualitative Comparative Analysis (fsQCA) and Necessary Condition Analysis (NCA), Tan and Wei (27) found that pharmaceutical firms can improve their overall ESG performance by strategically allocating resources and capabilities while considering ESG performance and financial leverage, among other factors, to improve total factor productivity. Based on Chinese A - share listed pharmaceutical manufacturing data from 2012 to 2021, Tan et al. (53) found that their firm ESG performance directly affects firm value.

Despite these valuable contributions, we identify an important gap in the literature. While existing research has established the importance of ESG performance and identified its key components, few studies have investigated concrete mechanisms for its improvement. Therefore, it is innovative and important for us to explore the effect of MAH on ESG performance of pharmaceutical manufacturing companies and its mechanism.

### 2.2 Policy background

In August 2015, China released *the Opinions on Reforming the Review and Approval System for Drugs and Medical Devices*, which for the first time suggested carrying out the pilot work of MAH (56). By November 2015, ten provinces and municipalities, Beijing, Tianjin, Hebei, Shanghai, Jiangsu, Zhejiang, Fujian, Shandong, Guangdong, and Sichuan, were selected for the three-year pilot program. In June 2016, China issued the *Notice on the Issuance of the Pilot Programme for the System of Holders of Listed Permits for Pharmaceuticals*, which clarified the scope of the pilot medicines, the conditions, obligations, and liabilities of each subject (54). In October 2018, China determined to extend the three-year pilot period of MAH by 1 year. In 2019, China enacted the newly revised *Drug Administration Law,* which clearly states that the state implements MAH for the administration of drugs (11).

The MAH system is characterized by three key innovations. Firstly, MAH broadens the scope of subjects of commissioned production, while enhancing the production efficiency of firms. MAH allows more types of subjects to participate in drug production, including R & D organisations that are not drug manufacturers, etc., which solves the problem of the scope of subjects in traditional entrusted production. The authorisation holder is legally responsible for the commissioned production firm, which allows the firm to have greater scale and resources for drug R & D and production. Secondly, MAH offers better protection for the intellectual property rights of R & D institutions and personnel. Holders of drug approval numbers can directly commission production to manufacturing enterprises that have obtained certification in line with the Good Manufacturing Practice (GMP) for pharmaceuticals. This measure provides R & D institutions with cutting - edge knowledge and technologies more room for development, enabling them to have greater control over the drug R & D and production processes. It also reduces intellectual property disputes arising from technology transfer. Third, MAH enhances the quality control of drug production. In contrast to the original system, MAH can alleviate the issue of information loss and distortion during the transmission process, thereby enhancing the quality and safety of drug production. Authorization holders are accountable for the entire supply chain and they have an incentive to guarantee the quality of their products. This also diminishes the ambiguity of risk and responsibility and boosts the effectiveness of quality management.

China has attained remarkable outcomes since the comprehensive implementation of the MAH system. According to the data from the *China Pharmaceutical Innovation and Research Development Association*,^1^ as of 30 November 2023, the number of B - license (Drug Manufacturing License Category B, which represents the holder of a marketing authorisation for commissioned production) firms in China had reached 1,172. With respect to regional distribution, aside from the ten centrally-administered provinces and municipalities where the pilot implementation was executed in 2015, Hainan, Hubei, Heilongjiang and other provinces have observed the most considerable increase in the number of B certificates.

### 2.3 Hypothetical development

Stakeholder theory states that business managers should understand and respect all individuals who are closely involved in the organization’s actions and outcomes, and try to balance and satisfy their interests as comprehensively as possible, rather than focusing only on the accumulation of shareholder wealth. According to this theory, the inclusion of various stakeholders in organizational decision - making is both a strategic resource and an ethical imperative, both of which contribute to the overall competitive advantage of the organization (28, 29).

The MAH builds a stakeholder system that includes listed company holders, contract - manufacturing companies and drug supervision and management authorities. Firstly, MAH enables the range of drug - listing license holders to be extended to drug R&D institutions and researchers, which promotes R&D incentives. Secondly, MAH allows the holder to entrust part or all of the drug production to other drug manufacturers. Thirdly, the governmental subjects of interest involved in MAH include the drug regulatory agency where the holder is located, the drug regulatory agency where the entrusted firm is located, and the State Drug Administration.

Stakeholders in pharmaceutical enterprises are concerned about environmental performance, such as the waste of raw materials and discarded packaging generated by the enterprises. A large amount of chemical waste generated during the production process of pharmaceutical enterprises, if discharged directly without effective treatment, will lead to the deterioration of the surrounding ecological environment, affect the quality of life of residents, and increase the cost of social environmental governance (30). Therefore, enterprises need to adopt green production technologies, enhance the utilization rate of raw materials, optimize packaging design, reduce waste generation, and achieve green and sustainable development to meet the expectations of the government and the public for environmental protection.

Stakeholders are concerned about the social performance of pharmaceutical enterprises. On the one hand, the public, as an important stakeholder, is highly concerned about whether enterprises can continuously innovate and provide innovative drugs that maintain social health (31). With the development of medical standards and changes in the disease spectrum, the public’s demand for innovative drugs is constantly increasing. Innovative drugs can bring better therapeutic effects to patients and improve the overall health level of society. On the other hand, as internal stakeholders of the enterprise, employees care about their own security during the process of drug research and development and production, including safety guarantees, salary guarantees, etc. (32). A safe working environment is the foundation for employees to work efficiently, and reasonable salary and benefits are the recognition of the value of employees’ labor, which directly affects their work enthusiasm and loyalty.

Stakeholders pay attention to the governance performance of pharmaceutical enterprises. Suppliers and customers, as stakeholders with close economic ties, expect to cooperate with enterprises that operate stably and have lasting competitiveness. A stable cooperative relationship is conducive to ensuring the smooth operation of the supply chain, reducing transaction costs, and achieving mutual benefit and win-win results. Investors and drug consumers will also make value judgments based on the financial performance and governance of enterprises, thereby influencing their investment decisions and consumption behaviors (33). A sound governance structure and financial status of an enterprise can enhance investor confidence and attract more funds to support its development.

Based on this, this study proposes the following hypotheses:

*H1*: MAH is conducive to promoting ESG performance in pharmaceutical manufacturing companies.

For the treatment of pharmaceutical pollution, it is better to strengthen front - end treatment than end - of - pipe treatment, as the removal of pharmaceuticals once they enter the environment will consume a lot of resources and present many technical difficulties. A more effective approach is to promote innovation in pharmaceutical production and to strengthen the management of pharmaceutical production, use and disposal. MAH protects the intellectual property rights of R & D organizations and personnel better, which helps to promote increased R & D investment by pharmaceutical manufacturing companies, reduce waste generation during the production of pharmaceuticals, and reduce the environmental pollution caused by pharmaceuticals during their use and disposal. Therefore, while MAH helps to reduce environmental pollution by promoting pharmaceutical innovation (34), it meets the regulatory needs of the government’s environmental protection department and responds positively to the concerns of international environmental organizations about environmental protection.

The government calls for quality and innovation in pharmaceuticals. In response, MAH will promote deeper specialization in the division of labor among pharmaceutical manufacturing companies and enhance their innovation capacity. R & D organizations will invest more resources in drug innovation, and manufacturing companies will be more committed to improving production efficiency and quality. Management will have to give up some of its profits to increase the salaries of production managers and R & D staff, thereby strengthening the quality management and innovation capacity of the firms. MAH will help pharmaceutical manufacturing firms to narrow the internal pay gap, and will also enhance the sense of belonging of their employees, which will promote their motivation in production and R & D, and bring sustained competitive advantages and economic benefits to the firms.

Besides, MAH may have a positive impact on the supply chain management of pharmaceutical manufacturing firms. On the one hand, in the traditional model, pharmaceutical manufacturing firms are responsible for both R & D and production, and the entire supply of pharmaceuticals will be seriously affected in the event of any problems in the firms, such as production equipment failure, quality control problems, and broken capital chain. MAH allows the holder to entrust different firms to produce. This provides pharmaceutical manufacturing firms with the possibility of reducing risks through supply chain management and promotes more efficient and safer pharmaceutical R & D and manufacturing. On the other hand, with the advancement of specialized division of labor and R & D innovation, pharmaceutical manufacturing firms in each segment will significantly improve their core competitiveness, increasing their competitive advantages in the face of suppliers and customers. This helps firms reduce the concentration of the supply chain and further diversify business risks (35).

*H2*: The channels through which MAH promotes ESG performance in pharmaceutical manufacturing firms are increasing R & D investment, reducing internal pay gaps, and reducing supply chain concentrations.

To verify these hypotheses, we constructed the DID model to implement the strategy of hypothesis testing. The acceptance or rejection of a hypothesis depends on the statistical significance and sign direction of the coefficients of the key variables in the model. All tests were conducted using two-sided tests, and the significance levels were set at 0.1, 0.05, and 0.01. The following text specifically describes the settings of the model and variables.

## 3 Methodology

### 3.1 Model and variables

We take China’s implementation of MAH in 10 pilot regions as an exogenous policy shock and employ the DID model to evaluate its influence on the ESG performance of pharmaceutical manufacturing firms. The DID model has been extensively utilized in the assessment of the impacts of the pilot policies, and it can effectively relieve the endogeneity problem in empirical studies (36). The benchmark regression equation is shown below:


(1) $$\mathrm{ES}G_{i,t+1}=\alpha +{\text{βTime}}_{t}\times {\text{Treat}}_{i}+\gamma X_{i,t}+\delta_{i}+\mu_{t}+\varepsilon_{i,t}$$

In Equation 1, i represents firms and t represents years. *δ* is an individual fixed effect, *μ* is a year fixed effect, and *ε* is a random disturbance term. We lag Treat×Time and all control variables by one period to further mitigate the effects of endogeneity.

Regarding ESG as an explanatory variable, referring to Li and Zhu (55), we first use ESG rating data from Bloomberg as a proxy variable. Bloomberg began to gradually establish an ESG rating system and conduct scoring since 2009. It covers thousands of listed enterprises worldwide, integrates data on environmental (E), social (S), and corporate governance (G) aspects, and assesses the ESG performance of enterprises through standardized scores (0–100), with higher scores indicating better performance. Its greatest advantage is that it takes into account the characteristics of the industry. For the pharmaceutical industry, it takes into account environmental aspects such as the traceability of raw materials by pharmaceutical enterprises and R&D investment to reduce the use of toxic reagents; social aspects such as the protection against exposure to high-risk chemicals by laboratories and production personnel; and corporate governance issues such as intellectual property rights and supply chains.

Besides, studies on ESG performance usually use ESG rating data from Sino-Securities Index Information Service (Shanghai) Co. Ltd. Later, we use this ESG rating data as a proxy variable for the robustness test.

Treat × Time is the explanatory variable that calculates the average effect of MAH on the ESG performance of pharmaceutical manufacturing enterprises in the pilot area. And deem the pilot implementation of MAH in China as a quasi - natural experiment. Group dummy variable; it gets the value of 1 if the enterprise’s registration is in the pilot area of MAH, and 0 otherwise. Time dummy variable; it attains the value of 1 if the year is after the implementation of MAH, and 0 otherwise.

We set Time as a dummy variable based on the year around 2016 for two reasons. First, the MAH pilot program was proposed only in November 2015, which was already the end of the year. Moreover, it usually takes some time for enterprises to carry out research and development and strategic challenges. Therefore, it is very likely that enterprises will start to adjust their strategies some time after the end of 2015. Secondly, in November 2015, China merely proposed to conduct a pilot program, but the implementation methods and key points were not clarified. It was not until June 2016 that the relevant content was determined. Besides, our approach is also in line with that of many other scholars (37). Therefore, we take 2016 as the starting year for the implementation of the MAH pilot.

X denotes a set of control variables which might exert an influence on the ESG performance of firms. In order to regulate the impact of other firm - and region - specific traits on the ESG performance of firms, we introduce the following control variables, including the ltime (the number of years since listing), the size of the firm, financial leverage (lev), profitability level (roa), the number of board of directors (board), the independence of the board of directors (ind_r), shareholding concentration (top1), the nature of property rights (soe), regional economic level (grp), and regional industrial structure (industry) (38). The specific definitions are exhibited in Table 1.

**Table 1:** Variable definition.

Notation	Name	Measurement
ESG	Firm ESG performance	See 3.1
Treat×Time	MAH system	See 3.1
ltime	Number of years listed	Natural logarithm of the number of years since the firm was listed (ln)
size	Firm size	total assets (ln)
lev	Financial leverage	Total liabilities/total assets (%)
roa	Profit level	Net profit/total assets (%)
board	Number of Board of Directors	Natural logarithm of the number of board members (ln)
ind_r	Board independence	Number of independent directors/number of board of directors (%)
top1	Shareholding concentration	Shareholding ratio of the largest shareholder (%)
soe	Nature of property rights	A value of 1 is assigned if the firm is state - owned, and 0 otherwise
grp	Regional economic level	Regional GDP (ln)
industry	Regional industrial structure	Percentage of GDP in the secondary sector (%)

### 3.2 Data sources

We selected Chinese A - share - listed pharmaceutical companies from 2012 to 2019 for the study. Further, following the general practice, we excluded sample data of companies with ST, ST* designations and a gearing ratio greater than 1. Eventually, this study obtained 81 sample companies with a total of 491 observations.

We chose 2012–2019 as our sample period because, first, MAH was fully implemented in China from 2020 onwards (11). On 1 December 2019, China started to implement a revised version of the *Drug Administration Law*, which has a special chapter on ‘marketing authorisation holders of drugs’, and in January 2020, it reviewed and passed the *Measures for the Administration of Drug Registration*, which came into effect from July 2020 onwards. Second, starting in 2020, China suffered the full brunt of COVID - 19. In 2020 and beyond, people’s health was under great threat, and there was a significant impact on pharmaceutical companies. By setting the end of the sample period in 2019, we were able to evade the impact of COVID - 19 on the study. Third, China’s MAH was implemented on a pilot basis from 2016, and we set the first period of the sample to 2012, which ensures the symmetry of the sample period.

Data on firm ESG performance are from Bloomberg, and data on other financial indicators are from the CSMAR database. Data at the regional level are from the *China Statistical Yearbook*. We processed the data using STATA17.

## 4 Empirical analysis

### 4.1 Descriptive statistics

The descriptive statistics of the main variables are presented in Table 2. Throughout the sample period, the mean value of ESG performance for listed companies amounts to 30.013, with the standard deviation being 9.479, the minimum value standing at 10.744, and the maximum value reaching 67.206. This suggests that the ESG performance within the sample exhibits a relatively significant disparity, which holds value for its study. At the same time, the mean value of Treat × Time is 0.364, signifying that the pilot firms possess 36.4 per cent of observations subsequent to the implementation of the MAH system, making it highly appropriate for the application of the DID model.

**Table 2:** Descriptive statistics.

Variable	Obs	Mean	Std. Dev.	Min	Max
ESG	491	30.013	9.479	10.744	67.206
Treat×Time	491	0.364	0.482	0	1
Treat	491	0.624	0.485	0	1
Time	491	0.566	0.496	0	1
ltime	491	2.334	0.764	0	3.258
size	491	22.591	0.84	20.185	25.056
lev	491	0.299	0.156	0.025	0.697
roa	491	0.088	0.066	−0.364	0.34
board	491	2.16	0.175	1.609	2.708
ind_r	491	0.37	0.05	0.25	0.625
top1	491	0.362	0.152	0.068	0.891
soe	491	0.321	0.467	0	1
grp	491	10.223	0.794	6.553	11.587
industry	491	0.407	0.092	0.162	0.563

### 4.2 Benchmark regression analysis

#### 4.2.1 Parallel trend test

In order to verify whether the study satisfies the parallel trend assumption, we conducted a dynamic effects test (39), constructing the model as follows:


(2) $$\mathrm{ES}G_{i,t+1}=\alpha +\sum_{t=2012}^{2019}\beta_{t}T_{t}\times {\text{Treat}}_{i}+\gamma X_{i,t}+\delta_{i}+\mu_{t}+\varepsilon_{i,t}$$

In Equation 2, we substitute the dummy variable Time in model (1) with a series of dummy variables T representing each year from 2012 to 2019. We take 2015, the year prior to the implementation of MAH, as the base period to examine whether there is a significant disparity between the experimental group and the control group in terms of ESG performance before and after 2016.

**Figure 1:**
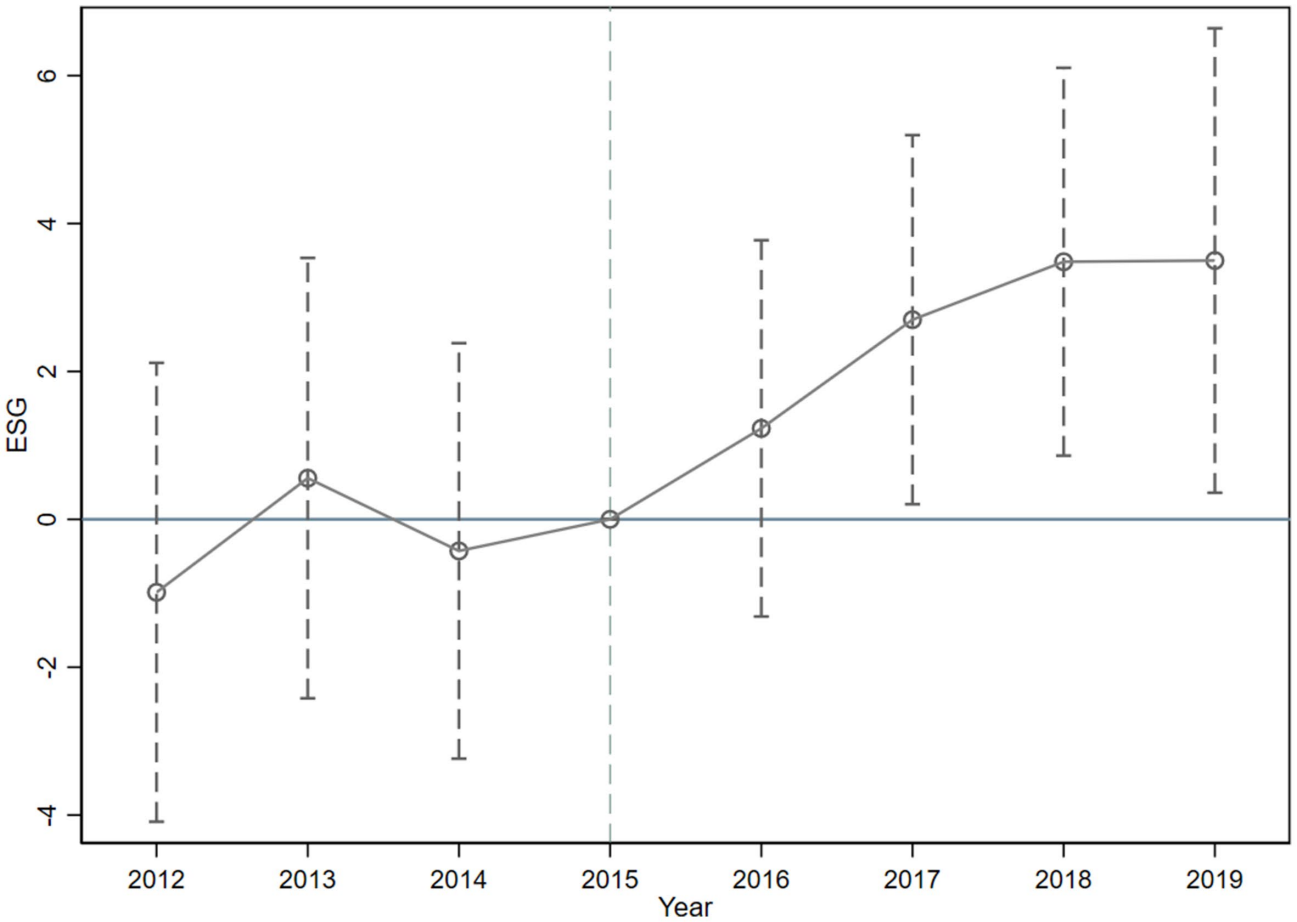
Dynamic effects plot.

In Figure 1, we mark the estimated coefficients of the dummy variables for each year as dots, and the dashed segments signify 95 per cent confidence intervals. The disparity in ESG performance between the pre - policy experimental group and the control group is insignificant, which implies that the study fulfills the parallel trend hypothesis. Meanwhile, we discover that the impact effect of the MAH regime is delayed by 1 year, which might be attributed to the long R & D cycle.

#### 4.2.2 Regression results

Table 3 shows the baseline regression results. We discover that, upon controlling for year fixed effects and individual fixed effects, the coefficient on Treat × Time is significantly positive at the 1 per cent level, irrespective of whether control variables are incorporated. Moreover, pharmaceutical manufacturing firms in the pilot region improved on average by 4.7% after 2016 (2.957*0.482/30.013), which is highly economically significant. This suggests that the MAH system contributes to the significant improvement of ESG performance of pharmaceutical manufacturing companies by constructing a stakeholder system of listed company holders, trustee manufacturers, and drug regulatory authorities.

**Table 3:** Baseline regression results.

	Without Control Variables	With Control Variables
	ESG	ESG
Treat×Time	2.933^***^	2.957^***^
	(0.753)	(0.746)
ltime		−1.598
		(1.011)
size		−0.924
		(0.848)
lev		0.591
		(3.218)
roa		11.776^***^
		(3.987)
board		−0.863
		(3.147)
ind_r		−0.848
		(8.915)
top1		2.951
		(5.322)
soe		1.644
		(1.337)
grp		4.546^*^
		(2.723)
industry		11.019
		(11.801)
_cons	28.996^***^	2.006
	(0.317)	(32.326)
Year FE	Yes	Yes
Individual FE	Yes	Yes
*N*	491	491
*R* ^2^	0.877	0.883

***, * indicate significant at the 1 per cent, 5 per cent and 10 per cent levels, with robust standard errors in parentheses.

The impact of the MAH system on the ESG performance of pharmaceutical manufacturing enterprises is specific. In terms of the environment, it can be reflected in the increased investment of enterprises in environmental protection measures. For instance, pharmaceutical enterprises can adopt more environmentally friendly raw materials and production technologies, reduce the generation and discharge of medical chemical waste, and increase investment in waste treatment facilities, thereby minimizing the impact on the surrounding ecological environment.

In terms of social performance, enterprises may invest more resources in research and development innovation, including increasing the recruitment and training of scientific researchers, establishing a more complete research and development incentive mechanism, and attracting more outstanding talents to participate in the research and development of innovative drugs. Meanwhile, enterprises pay more attention to the welfare and rights of R & D and production employees, increase salary and benefits, and improve the working environment to enhance employee satisfaction and loyalty.

In terms of corporate governance, enterprises may further improve their corporate governance structure and enhance the scientificity and fairness of decision-making. At the same time, enterprises may also enhance the transparency of information disclosure, promptly and accurately revealing their ESG performance and development strategies to investors and other stakeholders.

### 4.3 Robustness check

#### 4.3.1 Placebo test

To prevent the impact of MAH on the ESG performance of pharmaceutical manufacturing firms from being influenced by unobservable omitted variables, we carry out a placebo test (40). We repeated the aforementioned process 500 times. In each repetition, we randomly picked the same quantity of units as the treatment group in the total sample to constitute a dummy treatment group, and considered the remainder as the control group.

**Figure 2:**
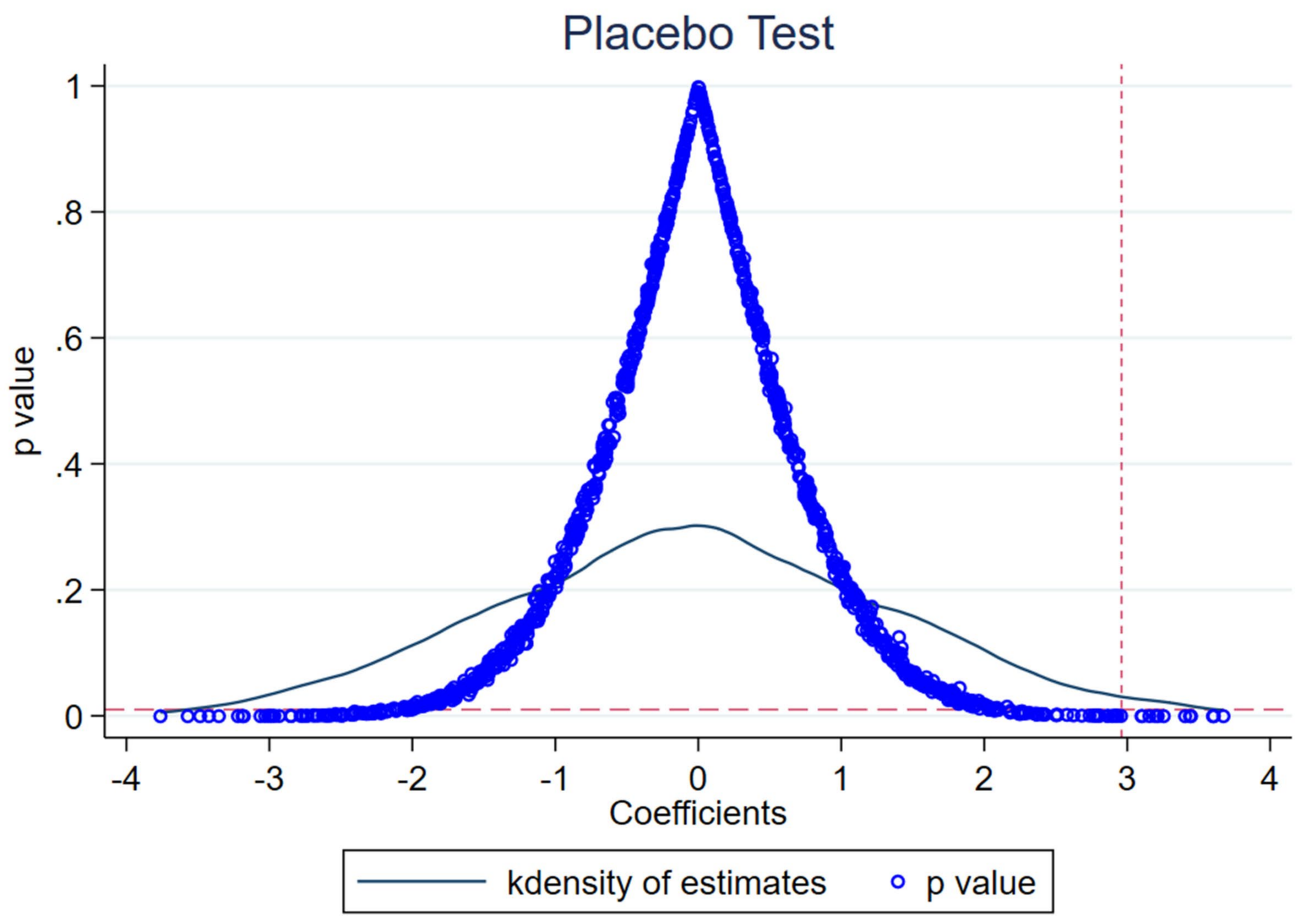
Placebo test.

The outcomes are presented in Figure 2, in which the majority of the estimated coefficients of the placebo test cluster around 0 and are insignificant, and the genuine estimated coefficients are outliers. This implies that the robustness of the baseline regression results, which constitutes the baseline conclusion, cannot be acquired following the placebo test for the randomized treatment group.

#### 4.3.2 Difference-in-Difference-in-Differences model test

Based on the DID model, we brought in a new set of experimental and control groups to build a Difference-in-Difference-in-Differences (DDD) model, which is beneficial for further removing the impact of potential confounding factors (41). The specific model is as follows:


(3) $$\begin{array}{l} \mathrm{ES}G_{i,t+1}=\alpha +{\text{βTime}}_{t}\times {\text{Treat}}_{i}\times \text{Medicin}e_{i,t}+{\text{θTime}}_{t}\\ \times {\text{Treat}}_{i}+{\text{ρTime}}_{t}\times \text{Medicin}e_{i,t}+{\text{σTreat}}_{i}\times \text{Medicin}e_{i,t}\\ +\text{φMedicin}e_{i,t}+\gamma X_{i,t}+\delta_{i}+\mu_{t}+\varepsilon_{i,t} \end{array}$$

In Equation 3 medicine serves as a dummy variable indicating whether firm i pertains to the pharmaceutical manufacturing industry in year t. If it does, it is assigned 1; otherwise, it is 0. The outcomes are presented in column (1) of Table 4. The estimated coefficients of the interaction term Treat × Time × Medicine in the DDD model are significantly positive at the 1% level, which validates the robustness of the findings.

**Table 4:** Robustness tests.

	DDD Model	PSM-DID 1:2	PSM-DID 1:4	Control the CDPPP	Measurement	Cluster Analysis
	ESG	ESG	ESG	ESG	ESG2	ESG
Treat×Time×Medicine	2.825^***^					
	(0.796)					
Treat×Time	0.179	4.924^**^	3.583^**^	2.920^***^	0.201^*^	2.957^**^
	(0.207)	(2.226)	(1.807)	(0.785)	(0.120)	(1.057)
Time×Medicine	−0.462					
	(0.546)					
Treat×Medicine	−5.823^***^					
	(1.721)					
Medicine	2.285^**^					
	(1.092)					
CDPPP				0.123		
				(0.930)		
_cons	8.305	2.053	−23.894	2.115	−0.511	2.006
	(6.049)	(51.663)	(48.982)	(32.465)	(4.772)	(34.370)
Controls	Yes	Yes	Yes	Yes	Yes	Yes
Year FE	Yes	Yes	Yes	Yes	Yes	Yes
Individual FE	Yes	Yes	Yes	Yes	Yes	Yes
*N*	7,496	157	225	491	491	491
*R* ^2^	0.855	0.928	0.909	0.883	0.705	0.883

***, **, and * represent significance levels of 0.01, 0.05, and 0.1, respectively. The values in parentheses in columns (1)–(5) are robust standard errors, and the value in parentheses in column (5) is the robust standard error clustered at the provincial level (53).

#### 4.3.3 PSM-DID test

Taking into account potential biases between the treatment and control groups that might impact the study results, we employed the propensity score matching (PSM) method to modify the sample (42). Firstly, we selected all control variables as matching variables. Secondly, we opted for the nearest - neighbor matching method to create a new dataset in two ratios of 1:2 and 1:4 respectively, in order to re - evaluate the effect of MAH on the ESG performance of pharmaceutical manufacturing enterprises.

The outcomes, as presented in columns (2) and (3) of Table 4, demonstrate that the positive impact of MAH on the ESG performance of pharmaceutical manufacturing enterprises is significant at the 5 per cent level at both scales, which aligns with the estimation of the baseline regression model and validates the robustness of the findings.

#### 4.3.4 Control the impact of the centralized drug purchasing pilot policy

When exploring the impact effect of a pilot policy, we usually need to consider the interference of the same type of policy. In December 2018, China launched the ‘4 + 7’ *Centralised Drug Purchasing Pilot Policy*. The policy is similar in timing to the MAH, and the policy has had a huge impact on the production and operation of pharmaceutical companies (43). Therefore, we use the DID model to generate its proxy variable CDPPP and include it as a new control variable in model (1).

The outcomes, as presented in column (4) of Table 4, indicate that the positive influence of CDPPP on the ESG performance of pharmaceutical manufacturing enterprises is insignificant. Meanwhile, the positive impact of MAH on the ESG performance of pharmaceutical manufacturing enterprises remains significant at the 1 per cent level, which further validates the robustness of the findings.

#### 4.3.5 Replacement of measurement

To examine the sensitivity of the measurement approach of the benchmark results, this paper substitutes the measurement method of the explained variable with the comprehensive score of Sino-Securities Index Information Service (Shanghai) Co. Ltd. ESG (44). As presented in column (5) of Table 4, following the replacement of the explained variable, the impact coefficient of the MAH on the ESG performance of pharmaceutical manufacturing enterprises remains significantly positive. This suggests that the benchmark conclusion is not susceptible to the measurement method of the explained variable.

#### 4.3.6 Cluster analysis

We are concerned that the treatment of standard errors may have influenced the benchmark conclusions. Since the MAH was piloted and implemented at the provincial and municipal levels in China, we grouped the standard errors at the provincial level where the enterprises are situated for analysis. As presented in column (6) of Table 4, the impact coefficient of the MAH on the ESG performance of pharmaceutical manufacturing enterprises remains significantly positive, which further illustrates the robustness of the research findings.

## 5 Expansion analysis

### 5.1 Mechanism testing

To test the three potential influencing mechanisms, we use the methods commonly employed in economic literature to construct the following model:


(4) $$\text{Mechanis}m_{i,t}=\alpha +{\text{βTime}}_{t}\times {\text{Treat}}_{i}+\gamma X_{i,t}+\delta_{i}+\mu_{t}+\varepsilon_{i,t}$$

In Equation 4, “Mechanism” stands for the three mechanism variables. Firstly, the R&D expense ratio is utilized to gauge R&D investment (Innovation). Secondly, the disparity between the average salary of the top three senior executives and the average salary of employees is computed, and then this value is divided by total assets to measure the internal wage gap (Wage_gap). We also measure it (Wage_gap2) by the ratio of the average salary of the top three senior executives to the average salary of employees to enhance robustness. Thirdly, we measure the degree of supply chain concentration from two aspects: the proportion of the largest customer (Consume) and that of the largest supplier (Vendor).

Table 5 presents the outcomes of the mechanism test. The MAH markedly enhances the R&D expense ratio of firms, narrows the internal wage gap, and reduces the proportion of the largest customer. However, the negative impact of the MAH on the proportion of the largest supplier is not significant. A possible reason is that some leading firms have emerged among the upstream suppliers of Chinese pharmaceutical manufacturing firms. These leading firms have grasped most of the orders in the market, including raw material suppliers, intermediate and excipient suppliers, as well as production equipment suppliers. Therefore, the change in the proportion of suppliers is relatively small.

**Table 5:** Mechanism testing.

	R & D Investment	Internal Pay Gap		Supply Chain Concentrations	
	Innovation	Wage_gap	Wage_gap2	Consume	Vendor
treat×time	0.011^***^	−0.006^***^	−0.835^*^	−0.038^***^	−0.020
	(0.003)	(0.002)	(0.433)	(0.011)	(0.017)
_cons	−0.024	0.847^***^	−103.577^***^	0.194	−1.426^*^
	(0.142)	(0.222)	(18.651)	(0.405)	(0.827)
Controls	Yes	Yes	Yes	Yes	Yes
Year FE	Yes	Yes	Yes	Yes	Yes
Individual FE	Yes	Yes	Yes	Yes	Yes
*N*	491	491	491	491	491
*R* ^2^	0.759	0.756	0.629	0.775	0.705

***, and * represent significance levels of 0.01, 0.05, and 0.1, respectively. The values in parentheses are robust standard errors.

### 5.2 Heterogeneity analysis

#### 5.2.1 Nature of property rights

We hold the view that the MAH system has a stronger ability to enhance the performance in terms of ESG of private enterprises as opposed to public enterprises. This is attributed to the fact that, on the one hand, private enterprises are more acutely responsive to the responses of the capital market. Consequently, they place greater emphasis on the performance in terms of ESG, which can yield substantial benefits from the capital market. On the other hand, in contrast to public enterprises that possess inherent advantages, private enterprises need to acquire enduring competitive edge through research and development innovation. Hence, the MAH system is more favorable for prompting private enterprises to enhance drug innovation.

As depicted in column (1) of Table 6, within the group of private enterprises, the estimated coefficient is markedly positive at the 1% level. As illustrated in column (2), in the group of public enterprises, the estimated coefficient is not significant. This validates our conjecture that the facilitating effect of MAH on the performance in terms of ESG of private enterprises is more evident.

**Table 6:** Heterogeneity analysis.

	Nature of property rights		Management ownership		Internal control	
	(1) non-SOEs	(2) SOEs	(3) less	(4) more	(5) lower	(6) higher
treat×time	4.475^***^	0.857	−0.760	2.720^***^	1.856	4.484^***^
	(0.936)	(2.004)	(2.235)	(1.000)	(1.538)	(1.048)
_cons	15.974	−44.597	−93.497	46.139	−20.638	−9.765
	(33.819)	(76.632)	(57.075)	(43.256)	(62.609)	(40.382)
Controls	Yes	Yes	Yes	Yes	Yes	Yes
Year FE	Yes	Yes	Yes	Yes	Yes	Yes
Individual FE	Yes	Yes	Yes	Yes	Yes	Yes
*N*	330	157	165	322	214	249
*R* ^2^	0.902	0.875	0.873	0.912	0.889	0.910

*** denote significance levels of 0.01, 0.05, and 0.1, respectively. The values inside the parentheses are robust standard errors.

#### 5.2.2 Management ownership

We consider that management ownership might serve as an efficient means to magnify the influence of the MAH. Owing to the presence of the agency issue, management typically refrains from taking risks in firm innovation. The setback of innovation has the potential to harm the management’s standing in the market, which explains why they frequently withdraw when confronted with innovation prospects. On the contrary, if the management possesses a larger share of stocks, they will attach greater significance to the long-term growth of the enterprise and augment investment in research, development, and innovation.

As indicated in column (3) of Table 6, within the group where the management ownership ratio is below the median, the estimated coefficient is insignificant. As demonstrated in column (4), in the group with a management ownership ratio no less than the median, the estimated coefficient is notably positive at the 1% level. This implies that the method of management ownership can motivate pharmaceutical manufacturing companies to engage more actively in the MAH, thereby markedly enhancing their ESG performance.

#### 5.2.3 Internal control

We hold the view that enhancing a company’s internal control could also be one of the effective approaches to motivate the company to engage in the MAH system. The degree of internal control dictates a company’s operational and management effectiveness. Companies with a superior level of internal control are inclined to place more emphasis on the long-term benefits of the company. Consequently, a company’s internal control might exert an influence on its R&D strategy, salary allocation, and supply chain management.

We employ the firm internal control index from the DIB database as a metric. The greater the index, the higher the level of internal control (45). As depicted in column (5) of Table 6, within the group with a lower internal control level (below the median), the estimated coefficient is insignificant. As illustrated in column (6), in the group with a higher internal control level (not less than the median), the estimated coefficient is notably positive at the 1% level. This suggests that internal control profoundly impacts the strategic decisions of pharmaceutical manufacturing firms and affects the connection between MAH and firm ESG performance.

### 5.3 Additional tests

#### 5.3.1 Variations in ESG performance

We investigated the effect of MAH on the disparities in ESG ratings among pharmaceutical manufacturing enterprises. Firstly, we computed the Bloomberg ESG^2^ and Sino-Securities Index Information Service (Shanghai) Co. Ltd. ESG^3^ variations. This was achieved by deducting the sample mean from each ESG value and subsequently dividing by the standard deviation. The discrepancy between these two computed values was employed to gauge the variations in firm ESG ratings (46). As illustrated in column (1) of Table 7, it was discovered that MAH did not exert a substantial influence on the differences in ESG ratings of pharmaceutical manufacturing firms.

**Table 7:** Additional analyses.

	Impression Management	Earnings Management
	ESG performance differences	Real earnings management
treat×time	−0.008	−0.031
	(0.201)	(0.035)
_cons	0.653	−0.808
	(9.061)	(1.714)
Controls	Yes	Yes
Year FE	Yes	Yes
Individual FE	Yes	Yes
*N*	491	491
*R* ^2^	0.643	0.850

***, **, and * represent significance levels of 0.01, 0.05, and 0.1, respectively. The figures in parentheses are robust standard errors.

#### 5.3.2 Real earnings management

Real earnings management reflects a company’s accounting information quality and earnings motivation. Generally speaking, when a company faces significant performance pressure, its management has a stronger incentive to implement real earnings management. Also, it is more likely to conduct impression management (the act of using false ESG disclosures to create a positive but potentially misleading image) through false ESG disclosures (47). As shown in column (2) of Table 7, we found that MAH also did not have a significant impact on the level of real earnings management of pharmaceutical manufacturing firms.

In recent years, there have been increasing doubts about companies using ESG for impression management (48). To address such concerns, we conducted the above two tests to explore whether the impact of MAH on the ESG performance of pharmaceutical manufacturing firms stems from impression management. The above results indicate that the firms affected by MAH do not have the motivation to conduct impression management through ESG disclosures.

## 6 Discussion

Compared with the existing research, the conclusion of this study shows unique theoretical value and practical significance. Regarding the impact of MAH on enterprises, previous studies have mostly focused on the single dimension of MAH on enterprise innovation or market performance. For instance, Liu et al. (11) pointed out that MAH has a significant positive impact on the innovation quality of the pharmaceutical manufacturing industry by increasing R&D investment. Wan et al. (12) found that the separation of marketing authorization and production authorization can promote pharmaceutical innovation, but few studies have approached it from the comprehensive perspective of ESG. This study found that MAH has a significant positive impact on the ESG performance of pharmaceutical manufacturing enterprises. It not only expands the research boundary of the economic consequences of the MAH system, but also confirms the key role of system innovation in promoting the sustainable development of enterprises.

From the perspective of institutional innovation, the MAH system promotes systematic improvement of enterprises in the dimensions of environment, society and governance by reconstructing the stakeholder system, including marketing authorization holders, entrusted manufacturing enterprises and regulatory authorities. This conclusion echoes the theory of institutional gaps in emerging economies (49), that is, in a market environment where the system is not yet perfect, institutional innovation can fill the gaps in regulation and resource allocation. MAH breaks the traditional “R&D - production binding” model and achieves the optimal allocation of resources through contract manufacturing, prompting enterprises to incorporate ESG goals into their strategic planning. This confirms the theory proposed by North (50) that “institutions promote economic development by reducing transaction costs and shaping incentive mechanisms,” indicating that emerging economies can guide the transformation of enterprise behaviors toward a sustainable direction through institutional innovation.

Enterprise heterogeneity analysis further reveals the mechanism of MAH. In non-state-owned enterprises, enterprises with high management holding shares and enterprises with good internal control, the improvement effect of MAH on ESG performance is more significant. This conclusion is highly consistent with the theory of ownership structure in emerging economies (51). Compared with state-owned enterprises, non-state-owned enterprises are more responsive to institutional changes due to their shorter decision-making chains and higher market sensitivity. Managing high-holding enterprises directly link ESG goals with management incentives by strengthening the binding of interests. In addition, a sound internal control system helps enterprises efficiently implement ESG strategies and reduce the friction costs of system implementation. These findings provide a theoretical basis for how enterprises with different owner-ownership and governance structures in emerging economies can maximize institutional dividends.

Furthermore, this study confirmed that there is no ESG performance greenwashing phenomenon in pharmaceutical manufacturing enterprises, which contrasts with the research conclusion that some industries “obtain policy dividends through superficial ESG behaviors” (52), and indirectly reflects the constraining effect of the strong regulatory nature of the pharmaceutical industry on the behavior of enterprises.

## 7 Conclusions, recommendations and limitations

### 7.1 Conclusion

Health issues have turned into a global concern. Especially after the eruption of COVID - 19, how to deal with sudden global diseases has become a central focus for all nations. Pharmaceutical manufacturing companies, as vital entities in protecting human health, urgently need scholars’ profound research on ways to boost their innovation and high - quality development. China initiated the piloting of the Marketing Authorization Holder (MAH) system in 2016 and fully implemented it in 2020. Based on the data of Chinese A - share listed pharmaceutical manufacturing enterprises from 2012 to 2019, we employed the DID model to examine the influence of MAH on the firms’ ESG performance, along with its underlying mechanisms and boundary conditions.

We discovered that, firstly, MAH has a markedly positive effect on the ESG performance of pharmaceutical manufacturing companies. Secondly, MAH drives the R&D investment of pharmaceutical manufacturing companies, narrows the internal salary gap within firms, and decreases the concentration of firm supply chains, thereby enhancing ESG performance. Thirdly, the impact of MAH is more evident in non - state - owned companies, companies with a higher proportion of management shareholding, and companies with a higher level of internal control, leading to a greater enhancement in ESG performance. Fourthly, MAH has no significant impact on the disparities in firm ESG ratings and real earnings management. There is no evidence of “greenwashing” in terms of ESG performance or the motivation for such behavior among pharmaceutical manufacturing companies.

### 7.2 Policy and management recommendations

#### 7.2.1 Policy recommendations

For China, based on the MAH system, the government ought to further introduce supportive policies to stimulate innovation in pharmaceutical manufacturing enterprises. Firstly, the government can offer assistance like tax incentives, R&D subsidies, and innovation awards to pharmaceutical manufacturing enterprises with outstanding innovation performance. This can lower their innovation costs and risks, and boost their enthusiasm and quality of innovation. Secondly, the government can help pharmaceutical manufacturing enterprises with good innovation performance in expanding their financing channels. The government can actively guide financial institutions to specially create financial products and services to encourage innovation in pharmaceutical manufacturing enterprises, such as intellectual property pledge financing and equity financing. Thirdly, the government is required to enhance the intellectual property protection mechanism in the field of pharmaceutical research and development. The government should establish a sound and reasonable system to protect drug innovation, safeguard the legitimate rights and interests of intellectual property owners, and promote healthy market competition.

We also recommend that the government should guide pharmaceutical manufacturing firms to actively fulfill their social responsibilities. Besides the research and development of innovative drugs, drug quality is also one of the core factors affecting the sustainable survival and development of pharmaceutical manufacturing firms. Firstly, the government can leverage the MAH system to encourage pharmaceutical manufacturing firms to enhance their efforts in attracting and cultivating innovative talents, as this is an internal driving force directly influencing the firms’ sustainable innovation. Secondly, the government should strengthen drug quality management and improve relevant systems. Through measures such as supervision and punishment, the government can enhance firms’ attention to and investment in drug quality. Thirdly, the government can streamline the social supervision and management mechanism. By setting up hotlines and mailboxes for reporting, the government can strengthen public supervision over pharmaceutical manufacturing firms and encourage the reporting of their improper behaviors.

In addition to this, we suggest that the government should optimize the supply chain of pharmaceuticals from R&D to manufacturing and distribution in a number of ways. Firstly, we suggest that the government can establish a comprehensive supplier information platform for the pharmaceutical industry. The government can integrate raw material suppliers, intermediates and excipients suppliers, and production equipment suppliers and other types of information, including product quality, price, capacity, reputation, etc., to provide detailed supplier information resources for pharmaceutical manufacturers, reducing the degree of information asymmetry. The government can also use the platform for dynamic monitoring of the operating conditions, reputation and quality of suppliers of pharmaceutical manufacturing firms, and timely release of relevant information and early warning signals. Second, the government should strengthen the macro-control of the pharmaceutical R&D and manufacturing market to guide healthy competition. In particular, the government needs to pay real-time attention to market monopoly behavior, and once firms are found to have gained or maintained an excessive market share through improper means, such as abusing a dominant market position and engaging in price monopoly, the government should investigate and punish them in a timely manner.

For other countries, the conclusions of this study also have certain reference value for their policy systems. For countries like Europe that have implemented the MAH system in the past, first of all, they can promote the establishment of a cross-border MAH regulatory collaboration mechanism. For instance, these countries can promote the standardized sharing of MAH data among EU member states, including drug safety data, credit records of holders, etc. Secondly, establish and improve the full life cycle liability insurance for MAH. The government can collaborate with insurance companies to develop differentiated insurance products, such as high-risk and high-coverage innovative drugs and low-premium generic drugs, to diversify the risks of the holders.

For some countries that have not yet implemented the MAH system, we suggest that these countries can give priority to piloting the limited MAH system in the field of generic drugs, allowing domestic research and development institutions to act as the holders and entrust local branches or regional cmos of multinational pharmaceutical companies to produce. In addition, these countries can cooperate with the international patent pool to authorize local enterprises to produce specific drugs whose patents have expired. The MAH qualification will only be granted to enterprises that commit to local supply, and regulatory personnel will be trained simultaneously.

#### 7.2.2 Management recommendations

We advise pharmaceutical manufacturing companies to strengthen R & D and cooperation. First, pharmaceutical manufacturing firms should enhance cooperation with other enterprises in R & D. Firms with technological and equipment advantages can cooperate with those having market and channel advantages. This cooperation can accelerate the process of new drugs from R & D to sales, and also give full play to their comparative advantages. Meanwhile, domestic firms can establish a cooperation with internationally renowned pharmaceutical firms. By doing so, they can introduce advanced foreign technology and management experience, participate in international drug R & D projects and standard - setting, thus enhancing the visibility and competitiveness of domestic pharmaceutical manufacturing firms in the international arena. Secondly, we suggest that pharmaceutical manufacturing firms strengthen their cooperation with universities and research institutes. Firms can integrate the resources of all parties to establish an industry - university - research platform. They can combine the financial and business advantages of state - owned firms with the R & D resources of scientific research institutes to jointly carry out major drug innovation projects, improving the efficiency of innovation.

We also urge that pharmaceutical manufacturing firms formulate a reasonable compensation system and other incentive measures. Firstly, to fully leverage the institutional advantages of MAH, pharmaceutical manufacturing firms must increase the compensation and other incentives for R & D personnel. Only by attracting, retaining, and motivating R & D talents can these firms ensure a strong innovation impetus. Secondly, apart from R & D personnel, firms must ensure and continuously improve the salary guarantees for front - line production workers. Production workers play a crucial role in pharmaceutical manufacturing firms, and their attitudes directly determine the quality of drugs. Based on meeting their basic needs, firms should use various incentive measures to enhance their sense of identity and belonging. Coupled with strict production management systems, this can ensure that drug production quality meets the standards.

In addition, we suggest that pharmaceutical manufacturing firms strengthen their supply chain management. Firstly, although we have not found that MAH has a significant impact on the supplier concentration of pharmaceutical manufacturing firms, we recommend that these firms enhance their management of suppliers. Pharmaceutical innovation carries high risks. Once suppliers encounter issues such as raw material shortages, contract disputes, or quality problems, purchasers will face significant production and operational risks. Furthermore, we suggest that pharmaceutical manufacturing firms appropriately strengthen their management of customers. Every link, from R & D to production and then to sales, is of vital importance. Pharmaceutical manufacturing firms should not only actively respond to market demands by researching and developing targeted new drugs but also increase promotional efforts based on genuine product features to enhance market competitiveness and promote continuous innovation.

### 7.3 Limitations

Our research has certain unaddressed limitations. Firstly, we primarily focused on the impact of MAH on the Chinese pharmaceutical market. In advanced countries such as those in Europe and America, MAH was implemented long ago and they have already entered the next stage. While our empirical evidence offers reference for many similar emerging countries, there is a lack of discussion on developed countries. Secondly, China fully implemented MAH after 2020, and some new issues emerged during this process. Meanwhile, after the COVID - 19 pandemic, global pharmaceutical research, development, and manufacturing have entered a new phase. We must admit that in order to respond to major sudden diseases, pharmaceutical - related systems (e.g., pharmaceutical regulatory systems) need further innovation. Relevant research should continuously pay attention to new systems. Thirdly, our research did not fully support the claim that MAH has a significant impact on the supplier concentration of pharmaceutical manufacturing firms. This conclusion may be affected by variable measurement and data quality. Perhaps subsequent research can provide evidence to support this view.

## Data Availability

The original contributions presented in the study are included in the article/supplementary material, further inquiries can be directed to the corresponding author.

## Footnotes

^1^ https://www.phirda.com/

^2^ https://www.bloombergchina.com/product/indices/

^3^ https://www.chindices.com/
